# Haptic versus conventional training in undergraduate implantology education: a randomized controlled pilot study

**DOI:** 10.3389/froh.2026.1938209

**Published:** 2026-09-28

**Authors:** Sandra Bosc, Amparo Aloy-Prósper, Maria-Angeles Fuster Torres, Jonathan P. San Diego, Rupert Austin, Berta García-Mira

**Affiliations:** 1 Faculty of Medicine and Dentistry, Department of Stomatology, Universitat de València, Valencia, Spain; 2 Doctoral Programme in Dentistry, Universitat de València, Valencia, Spain; 3 Faculty of Dentistry, Oral & Craniofacial Sciences, King's College London, London, United Kingdom

**Keywords:** dental education, haptic simulator, haptic virtual reality, implantology, preclinical training

## Abstract

**Introduction:**

Haptic virtual reality simulation is increasingly used in dental education as a complementary tool for preclinical training. In oral implantology, where implant placement requires precise control of depth, angulation, and spatial positioning, haptic simulation may help students acquire technical skills in a safe and repeatable environment. This pilot study aimed to evaluate the effectiveness of haptic simulation in undergraduate implantology training by comparing implant placement accuracy, procedural time, student satisfaction, motivation, and self-perceived improvement across different training modalities and sequences.

**Methods:**

A randomized, controlled, parallel-group pilot study was conducted with 68 third-year dental students enrolled in an Oral Surgery course. Participants were randomly allocated into four groups: Phantom Group (PG), conventional jaw-model training only; Simulator Group (SG), haptic simulation only; Mixed Group 1 (MG1), haptic simulation followed by conventional training; and Mixed Group 2 (MG2), conventional training followed by haptic simulation. Implant placement accuracy was assessed through coronal, apical, vertical, and angular deviations. Drilling time and total procedure time were recorded. Satisfaction, motivation, and self-perceived improvement after training were evaluated using five-point Likert-scale questionnaires. Non-parametric tests were used for inferential analyses, with statistical significance set at *p* < 0.05.

**Results:**

No statistically significant between-group differences were detected in coronal, apical, vertical, or angular deviations within either training modality. However, MG1 showed numerically lower coronal, apical, and angular deviations, particularly during conventional jaw-model training. No statistically significant between-group differences were detected in procedural times; however, MG1 showed the shortest drilling times in both modalities. Satisfaction and motivation were high across all groups. Statistically significant between-group differences were observed in selected items related to perceived realism and self-perceived improvement after training; however, these isolated findings should be considered exploratory given the multiple comparisons performed. No adverse events were reported.

**Conclusion:**

Haptic simulation was a well-accepted complementary tool for undergraduate implantology education. Although objective accuracy and procedural-time outcomes did not differ significantly between groups, exploratory differences in selected student-reported outcomes warrant further investigation of potential sequence-related effects. Larger studies with longer training exposure are needed to determine its effect on practical skill acquisition.

## 1 Introduction

In dentistry, **simulation-based learning** has become an essential tool for the education and training of students. Simulation is defined as a constructive learning strategy that promotes the acquisition of practical skills in a controlled and safe environment (1). Historically, the evolution of simulation in dental education has progressed from the use of human jawbones and plaster models with natural teeth, to acrylic replicas, and finally to the creation of the first phantom head by Oswald Fergus in 1894 (2). These tools have been foundational in preclinical education, enabling students to develop technical competence prior to engaging in patient care (3).

A key **advantage of simulation** is the ability to learn through trial and error without risk to patients. In the early stages of dental training, a significant portion of manual skills is acquired through simulated practice, allowing students to become familiar with clinical instruments, procedures, and ergonomics (3, 4). However, traditional simulators have limitations in replicating complex clinical scenarios, particularly those requiring fine motor control, real-time decision-making, and patient-specific adaptations (5).

One of the most significant innovations in this field has been the development of **virtual reality (VR) haptic simulators**, which integrate immersive virtual environments with tactile feedback (2, 6). These systems allow students to interact with virtual clinical scenarios while receiving sensory cues such as pressure, vibration, and auditory signals. Through sensory feedback, this type of system enhances realism and student engagement, while allowing repeated skill development without the ethical implications or unpredictability linked to live patient treatment (7). Haptic simulation may also support more sustainable preclinical education by reducing reliance on disposable materials and repeated physical models, in line with Sustainable Development Goals 4 and 12 (8, 9). In addition to increasing engagement and motivation, haptic simulation enables objective performance tracking, critical feedback, and deliberate practice—features essential to mastering complex procedures. For example, tasks such as implant placement require precise control of angulation and depth, skills that are difficult to cultivate using conventional plastic models. Traditional phantom-based simulators provide limited anatomical variability and do not generally offer automated real-time feedback on procedural errors (10).

Recent evidence reflects the increasing integration of immersive and haptic technologies into dental education. Global data indicate growing adoption of these technologies across dental institutions, while recent literature highlights their potential to provide standardized, repeatable, and interactive learning environments. Nevertheless, their optimal integration into existing preclinical curricula remains an important area of investigation (11, 12).

Previous studies have evaluated the effectiveness of haptic simulation across different areas of dental education. Farag et al. (13) conducted a prospective study in restorative dentistry, showing that students trained with haptic simulators performed significantly better in cavity design and overall execution time in real patients than those who used traditional methods. Similarly, Kikuchi et al. (14) found that students practising ceramic-metal crown preparation on virtual systems achieved superior outcomes in terms of accuracy and efficiency. More recently, Joseph et al. (15) examined the ability of haptic simulation to distinguish between different levels of experience in restorative dentistry, highlighting its potential role in objective skills assessment.

Joseph et al. (16) evaluated haptic simulation for implant surgery training in 40 third-year dental students and 20 experienced dentists. Students were randomized to theoretical instruction alone or theoretical instruction plus simulator training, after which all participants performed implant placement on a phantom model. Outcomes included positional and angular deviations, drilling depth, drilling and total procedure times, and cortical perforations. Simulator-trained students showed better accuracy than novices, with lower mean positional deviation (0.57 ± 0.06 mm vs. 0.85 ± 0.09 mm) and fewer cortical perforations (10% vs. 20%), approaching the performance of experienced clinicians (0.43 ± 0.05 mm) (16).

Zhang et al. (17) conducted a randomized controlled trial involving 80 s- and third-year dental students assigned to virtual simulation, jaw-model training, virtual followed by jaw-model training, or the reverse sequence. After theoretical instruction and an 8-hour practical session, outcomes included theoretical knowledge, clinician-rated operative performance, and CBCT-based implant accuracy measured through angular and linear deviations at the implant shoulder and apex. The combined-training groups achieved significantly better theoretical and practical performance and greater implant placement accuracy than the single-modality groups, with markedly lower angular deviation (approximately 1.77° vs. 5.85° for virtual training alone and 5.53° for jaw-model training alone) (17).

Despite promising findings, evidence on the use of haptic simulation in undergraduate implantology remains limited, particularly regarding the influence of training sequence. Implant placement requires a high degree of technical precision, and student motivation, confidence, and perceptions of skill development may also influence the learning process (3). Therefore, further studies are needed to determine how haptic and conventional training should be integrated within preclinical implantology curricula (4).

The present study aimed to compare the effects of haptic simulation, conventional jaw-model training, and two combined training sequences in undergraduate dental students. Implant placement accuracy was evaluated through coronal, apical, vertical, and angular deviations, while drilling time, total procedure time, satisfaction, motivation, and self-perceived improvement after training were assessed as secondary outcomes. We hypothesized that combined training would be associated with better implant placement accuracy, greater procedural efficiency, and more favorable student-reported outcomes than single-modality training. We further explored whether haptic simulation performed before conventional training was associated with more favorable objective and student-reported outcomes than the reverse sequence.

## 2 Methods

### 2.1 Study design

A randomized, controlled, parallel-group pilot study was conducted at the Department of Oral Surgery, University of Valencia, between October 2022 and May 2023. Ethical approval was obtained from the Ethics Committee of the University of Valencia (Registry No. 2490480), and all participants provided written informed consent prior to inclusion. Before the practical sessions, all participants completed a standardized teaching module consisting of four theoretical lectures on oral implantology. The study was designed to compare conventional jaw-model training, haptic simulation, and two combined training sequences.

### 2.2 Participants

Participants were third-year dental students enrolled in the Oral Surgery course at the University of Valencia. Eligible students were required to be enrolled in the course, to have attended the theoretical implantology sessions, and to provide written informed consent. Students who did not meet these criteria were excluded. Participation was voluntary.

### 2.3 Intervention

Participants were assigned to one of four groups:

- Phantom Group (PG): conventional jaw-model training only
- Simulator Group (SG): haptic simulation training only
- Mixed Group 1 (MG1): haptic simulation followed by conventional jaw-model training
- Mixed Group 2 (MG2): conventional jaw-model training followed by haptic simulation

This design allowed comparison of the training modalities and exploratory assessment of potential sequence-related differences (Figure 1).

**Figure 1:**
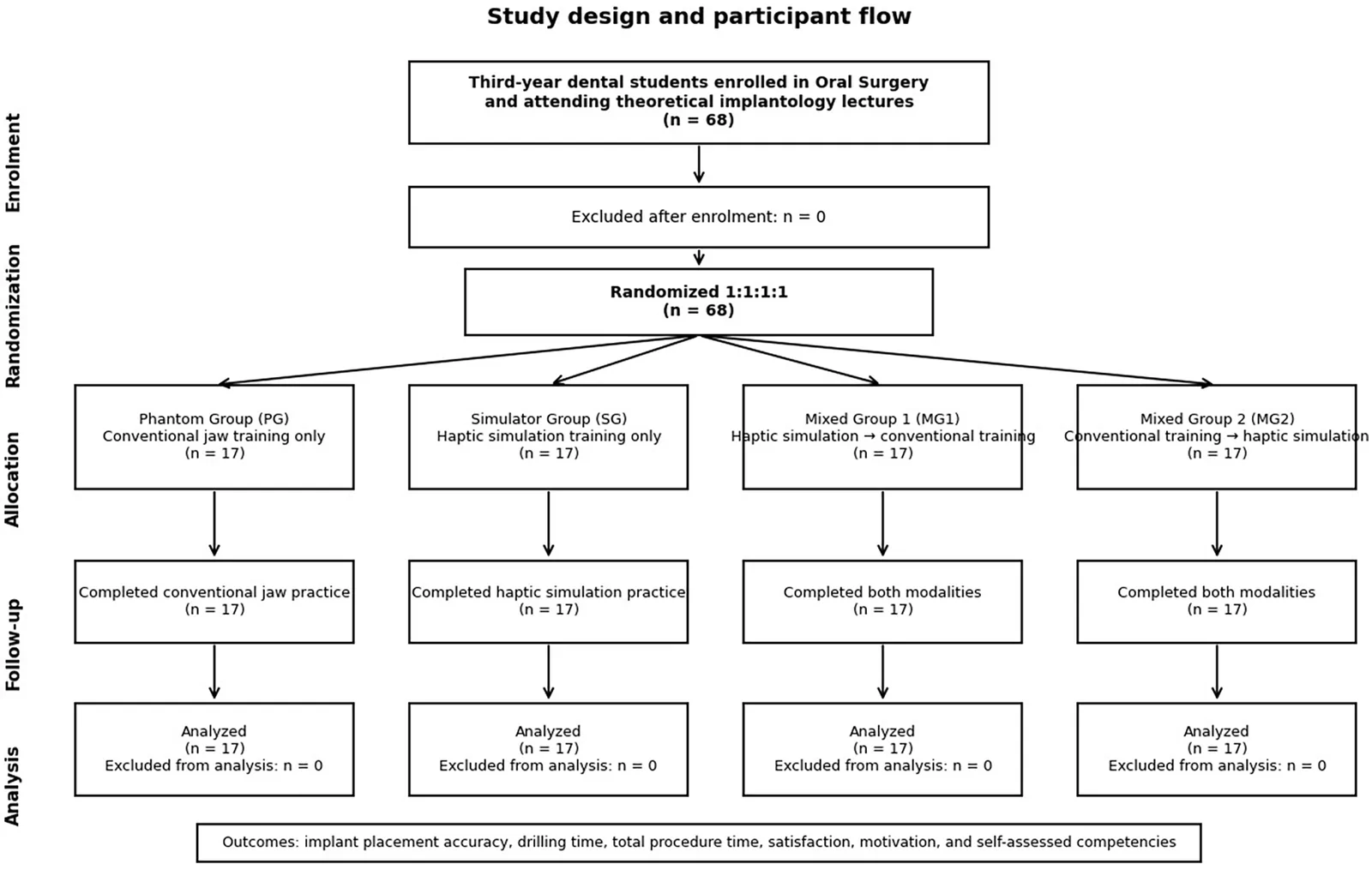
Study design and participant flow. Third-year dental students enrolled in the Oral Surgery course were randomized into four groups: Phantom Group (PG), Simulator Group (SG), Mixed Group 1 (MG1), and Mixed Group 2 (MG2). All participants completed the assigned training sequence and were included in the final analysis.

#### 2.3.1 Conventional jaw-model training

Before the practical exercise, students received a standardized procedural briefing supported by a PowerPoint presentation, covering incision design, flap elevation, and the drilling sequence. Students then performed a full-thickness flap elevation on phantom jaw models, followed by implant site preparation according to the manufacturer's recommended drilling protocol. Two implants measuring 4.0 mm in diameter and 10 mm in length (Galimplant, Sarria, Spain) were placed at sites 35 and 36 in each model (Figure 2). Each student performed the procedure once under the same instructional conditions.

**Figure 2:**
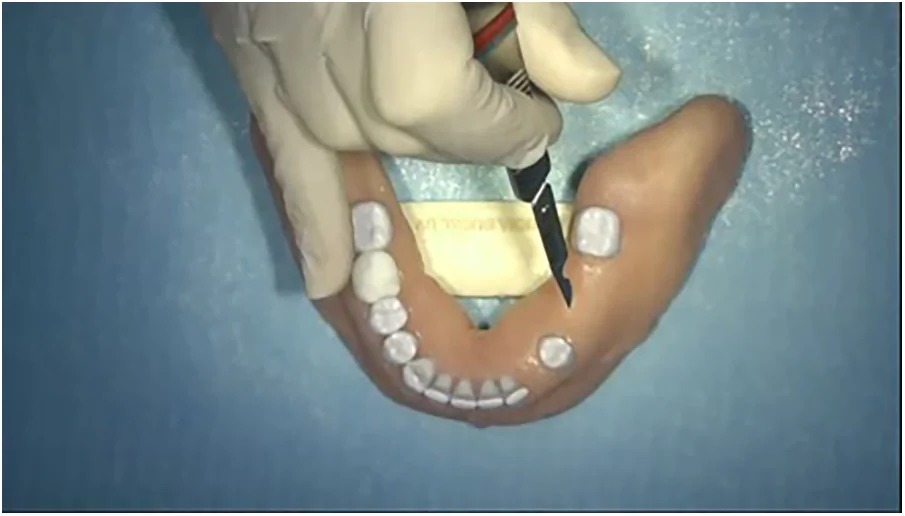
Phantom jaw model used in conventional implantology training.

#### 2.3.2 Haptic simulation training

Before the simulated exercise, students received a standardized briefing supported by a PowerPoint presentation on the use of the simulator and the procedural workflow. Students then practised on a virtual clinical case that replicated the anatomical characteristics of the conventional phantom jaw model, using the SIMtoCARE Dente® haptic training simulator (SIMtoCARE B.V., Vreeland, The Netherlands) (Figure 3).

**Figure 3:**
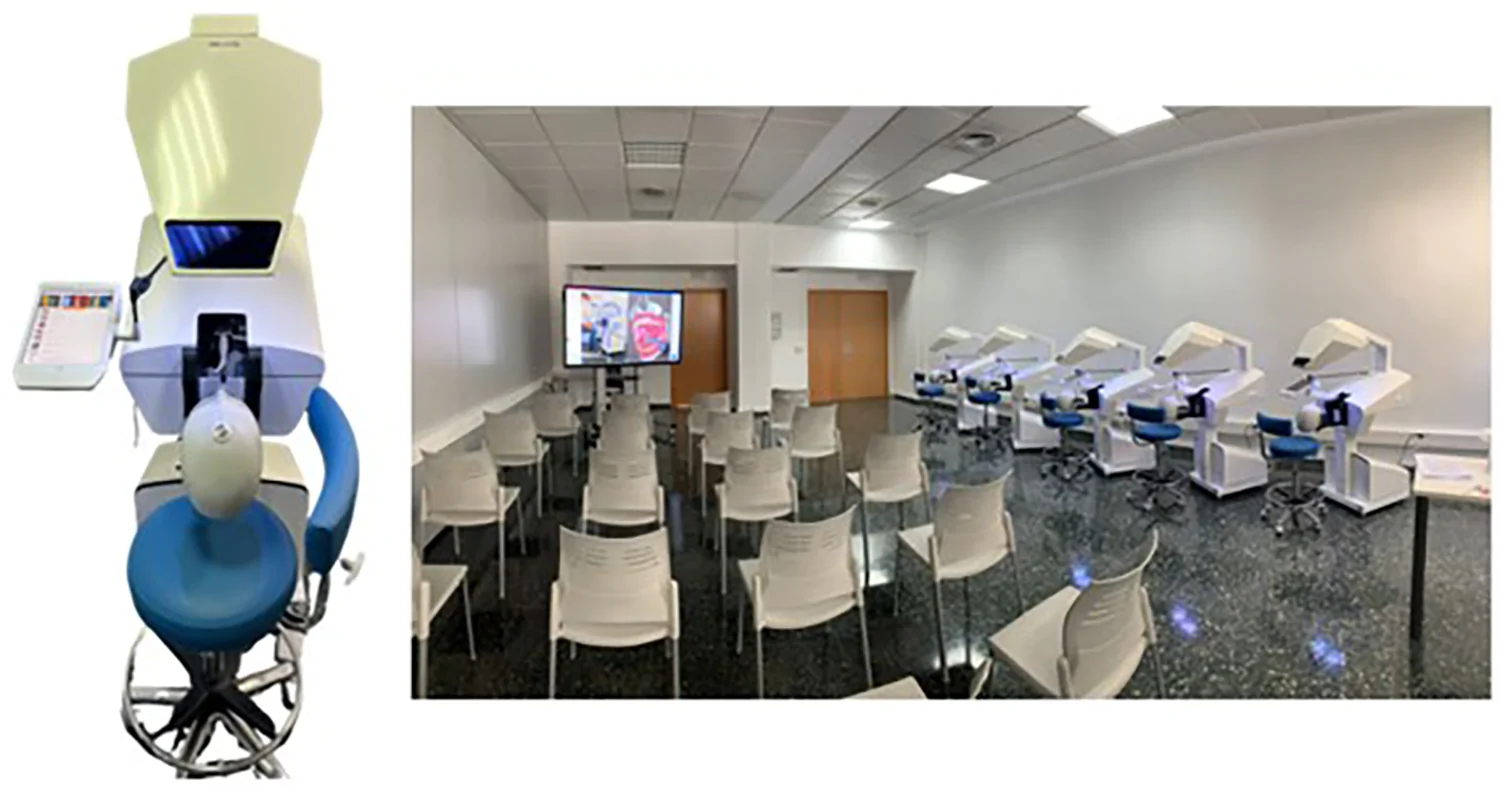
Haptic simulation room of the university of valencia.

The simulated case involved implant placement at sites 35 and 36 (Figure 4). Students completed standardized implant-placement exercises, and accuracy and procedural time data were automatically recorded by the simulator.

**Figure 4:**
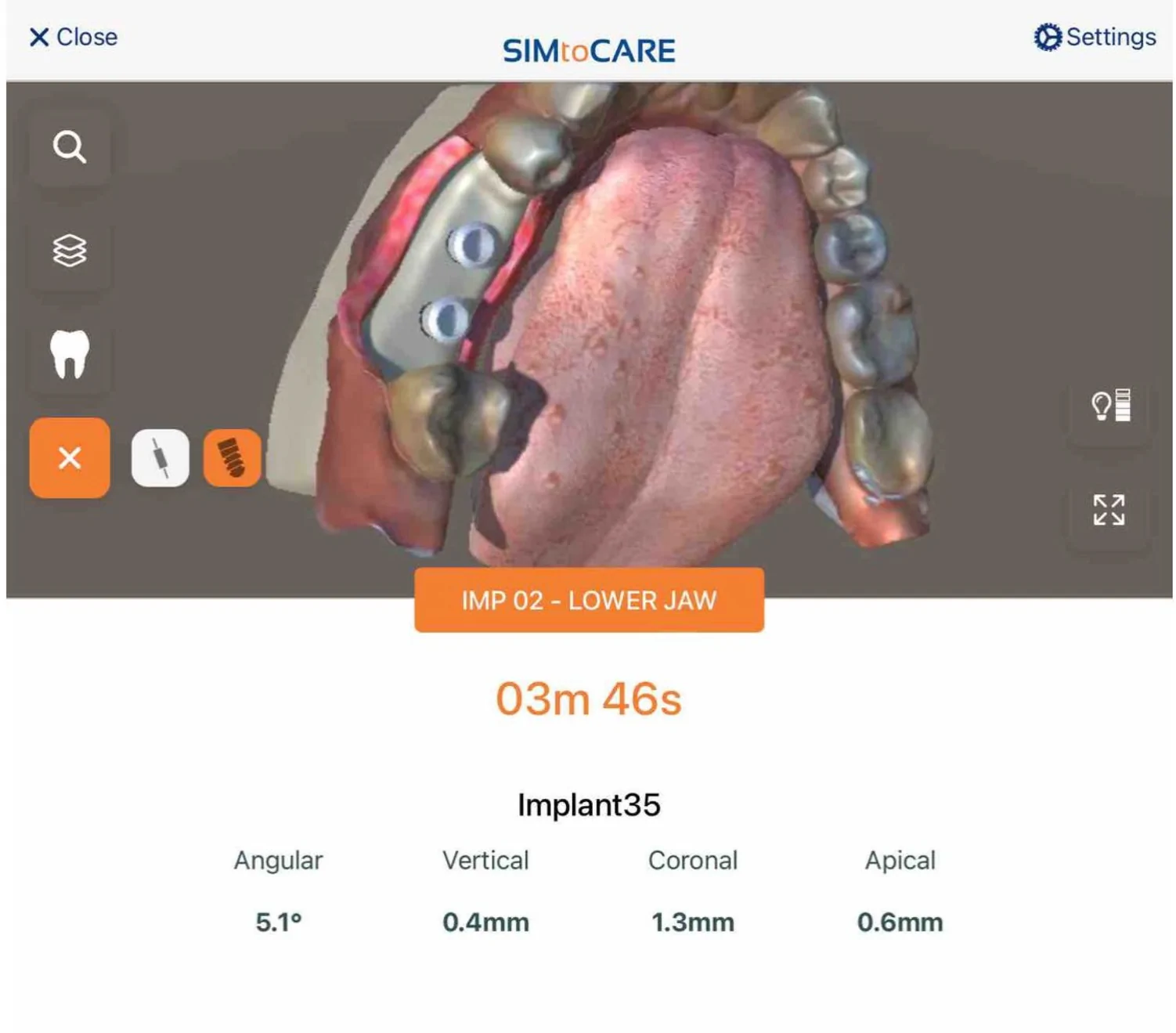
Haptic simulation training using the SIMtoCARE dente® dental simulator.

### 2.4 Outcomes

#### 2.4.1 Implant placement accuracy

Implant placement accuracy was the primary outcome and was assessed using four parameters: horizontal coronal deviation, horizontal apical deviation, vertical deviation, and angular deviation. For conventional jaw-model training, a reference cone-beam computed tomography (CBCT) scan (NewTom Giano HR, Imola, Italy) of a master model prepared by an experienced instructor was used to define the planned implant positions. After each student completed the procedure, a CBCT scan of the corresponding phantom was acquired. The student and reference models were superimposed using CoDiagnostiX software (Dental Wings, Montreal, Canada), and deviations between the planned and actual implant positions were calculated.

For haptic simulation, the same accuracy parameters were automatically generated by the SIMtoCARE Dente® simulator by comparing the planned and final implant positions.

Horizontal coronal and apical deviations and vertical deviation were expressed in millimetres, whereas angular deviation was expressed in degrees. Positive vertical deviation values indicated insufficient drilling depth, whereas negative values indicated over-drilling beyond the planned depth.

#### 2.4.2 Procedural time

Drilling time and total procedure time were recorded for both training modalities. During conventional jaw-model training, both variables were measured manually using a stopwatch. During haptic simulation, drilling time and total procedure time were automatically generated by the simulator. All time measurements were expressed in minutes.

#### 2.4.3 Satisfaction

The questionnaires were administered immediately after completion of the corresponding training modality. Student satisfaction was assessed after each training modality using structured questionnaires rated on a five-point Likert scale, ranging from 1 (“strongly disagree”) to 5 (“strongly agree”).

After conventional jaw-model training, participants in PG, MG1, and MG2 evaluated the adequacy and realism of the clinical case and the usefulness of the exercise for manual skill development.

After haptic simulation, participants in SG, MG1, and MG2 evaluated the realism of the virtual case, consistency with the theoretical content, interface usability, ergonomic suitability, and graphical quality.

#### 2.4.4 Motivation

Student motivation was assessed after each training modality using five-point Likert-scale questionnaires.

Following conventional jaw-model training, participants evaluated the perceived contribution of the session to their understanding of implantology, their interest in implantology training, their motivation to continue learning, and whether they would recommend the practical session to other students.

Following haptic simulation, participants evaluated its perceived educational value, contribution to understanding theoretical concepts, effect on motivation, and perceived relevance for future undergraduate cohorts.

#### 2.4.5 Self-perceived improvement after training

After each training modality, students rated their perceived improvement in six learning-related domains: theoretical knowledge, practical skills, critical thinking, motivation, confidence, and stress management. Responses were recorded on a five-point Likert scale ranging from 1 (“not at all”) to 5 (“significantly”).

The questionnaires were developed specifically for this exploratory study and reviewed by members of the teaching team for content relevance and clarity; no formal psychometric validation was performed.

The complete satisfaction, motivation, and self-perceived improvement questionnaires are provided in the Supplementary Material.

### 2.5 Randomization

Participants were randomly allocated in a 1:1:1:1 ratio to PG, SG, MG1, or MG2 using a predefined randomization sequence generated by the principal investigators. Group assignments were concealed in sequentially numbered, sealed, opaque envelopes, which were opened only after enrolment.

### 2.6 Blinding

Owing to the nature of the interventions, participants and instructors could not be blinded to the assigned training modality. Implant placement accuracy was assessed using objective digital measurements obtained through CBCT-based superimposition for conventional training and automatically generated simulator data for haptic training.

### 2.7 Sample size

This study included the complete cohort of 68 eligible third-year dental students who agreed to participate, with 17 participants allocated to each study group. As this was an exploratory randomized pilot study, no formal *a priori* sample size calculation was performed. The study was intended to provide preliminary estimates for the design of future confirmatory studies.

### 2.8 Statistical analysis

Descriptive statistics were calculated for all study variables. Categorical variables were summarized using absolute frequencies and percentages, whereas quantitative variables were summarized using the mean, standard deviation, range, median, and quartiles. Descriptive results were obtained for the overall sample and separately by study group. Student-reported questionnaire items measured using Likert scales were treated as ordinal variables; therefore, non-parametric methods were used for inferential analyses.

For student-reported outcomes assessed after conventional jaw-model training, pairwise between-group comparisons were performed using the Mann–Whitney U test among the three groups exposed to this modality: PG versus MG1, PG versus MG2, and MG1 versus MG2. For outcomes assessed after haptic simulation training, the corresponding comparisons were SG versus MG1, SG versus MG2, and MG1 versus MG2. These analyses were performed for the relevant satisfaction, educational, motivation, and self-perceived improvement items. The same three pairwise contrasts were applied to each questionnaire item within the corresponding training modality.

Direct within-participant comparisons between conventional jaw-model and haptic simulation training were performed for questionnaire items assessing equivalent constructs across the two modalities. The paired constructs were clinical case adequacy, perceived realism, contribution to understanding theoretical concepts, motivation to learn, usefulness for manual skill development, interest or educational value of the training, and willingness to recommend the training. The six self-perceived improvement domains were paired directly across modalities: theoretical knowledge, practical skills, critical thinking, motivation, confidence, and stress management. These correspondences reflected the item equivalences used in the original statistical analysis.

For participants who completed both modalities, these within-participant comparisons were performed using the Wilcoxon signed-rank test separately in MG1 and MG2; the two mixed-sequence groups were not pooled. Thus, conventional and haptic ratings were compared within MG1 and independently within MG2 for each of the corresponding questionnaire constructs described above. In MG1, the seven general, educational, and satisfaction-related paired items and the six self-perceived improvement domains were analyzed separately; the same procedure was applied independently in MG2.

Potential differences related to training sequence were explored through the MG1 versus MG2 Mann–Whitney U comparisons within each modality, together with the separate within-participant Wilcoxon analyses performed in MG1 and MG2. These analyses were exploratory and did not constitute a formal test of a modality-by-sequence interaction.

All statistical tests were two-sided, and statistical significance was set at *α* = 0.05. No adjustment for multiple comparisons was applied in the original exploratory analysis. Accordingly, statistically significant findings arising from the multiple pairwise comparisons were considered exploratory and hypothesis-generating and were interpreted cautiously because of the increased risk of type I error. Statistical analyses were performed using SPSS for Windows, Version 15.0 (SPSS Inc., Chicago, IL, USA).

## 3 Results

### 3.1 Participant flow

A total of 68 third-year dental students were enrolled and randomized, with 17 participants allocated to each group: Phantom Group (PG), Simulator Group (SG), Mixed Group 1 (MG1), and Mixed Group 2 (MG2). All participants completed the assigned training sequence, and no participants were excluded from the final analysis (Figure 1).

### 3.2 Accuracy

Implant placement accuracy outcomes are presented in Table 1. No statistically significant between-group differences were observed in horizontal coronal, horizontal apical, vertical, or angular deviation within either training modality (all *p* > 0.05). During conventional jaw-model training, MG1 showed numerically lower horizontal coronal deviation (1.35 mm), horizontal apical deviation (1.76 mm), and angular deviation (5.40°) than PG (1.58 mm, 2.11 mm, and 7.75°, respectively) and MG2 (1.60 mm, 1.99 mm, and 6.00°, respectively). During haptic simulation training, MG1 also showed numerically lower horizontal coronal deviation (1.55 mm), horizontal apical deviation (2.35 mm), and angular deviation (8.93°) than MG2 (1.78 mm, 3.00 mm, and 11.25°, respectively). None of these numerical differences reached statistical significance.

**Table 1:** Implant placement accuracy by training modality and group.

Type of practice	Groups	Horizontal coronal deviation (mm)	Horizontal apical deviation (mm)	Vertical deviation (mm)	Angular deviation
Conventional jaw-model training	PG	1.58	2.11	−0.56	7.75°
	MG1	1.35	1.76	−0.83	5.40°
	MG2	1.60	1.99	−0.63	6.00°
Haptic simulation training	SG	1.60	2.43	−0.05	9.50°
	MG1	1.55	2.35	0.40	8.93°
	MG2	1.78	3.00	0.03	11.25°

### 3.3 Procedural time

Procedural-time outcomes are presented in Table 2. During conventional jaw-model training, median drilling time was 3.87 min (IQR, 2.57–4.47) in PG, 3.10 min (IQR, 2.70–4.93) in MG1, and 3.47 min (IQR, 2.77–3.88) in MG2. Median total procedure time was 13.20 min (IQR, 11.98–16.30) in PG, 14.83 min (IQR, 12.83–17.50) in MG1, and 14.73 min (IQR, 11.47–16.67) in MG2.

**Table 2:** Procedural time by training modality and group. Values are presented as median (interquartile range).

Training modality	Group	Drilling time, median (IQR), min	Total procedure time, median (IQR), min
Conventional jaw-model training	PG	3.87 (2.57–4.47)	13.20 (11.98–16.30)
	MG1	3.10 (2.70–4.93)	14.83 (12.83–17.50)
	MG2	3.47 (2.77–3.88)	14.73 (11.47–16.67)
Haptic simulation training	SG	0.93 (0.65–1.22)	3.13 (2.36–4.13)
	MG1	0.84 (0.72–1.23)	2.98 (2.50–3.62)
	MG2	1.04 (0.72–1.45)	3.39 (2.69–4.61)

During haptic simulation training, median drilling time was 0.93 min (IQR, 0.65–1.22) in SG, 0.84 min (IQR, 0.72–1.23) in MG1, and 1.04 min (IQR, 0.72–1.45) in MG2. Median total procedure time was 3.13 min (IQR, 2.36–4.13) in SG, 2.98 min (IQR, 2.50–3.62) in MG1, and 3.39 min (IQR, 2.69–4.61) in MG2.

No statistically significant between-group differences were observed for drilling time or total procedure time within either training modality (all *p* > 0.05). MG1 showed the numerically shortest median drilling time in both modalities.

### 3.4 Satisfaction

Satisfaction ratings were high across both training modalities (Table 3). Following conventional jaw-model training, mean scores ranged from 3.82 to 4.88. Clinical case adequacy and usefulness for manual dexterity did not differ significantly between groups (all *p* > 0.05). Model realism differed significantly between PG and MG1 (*p* = 0.014), with higher ratings in PG (4.53 ± 0.72) than in MG1 (3.82 ± 0.81).

**Table 3:** Satisfaction outcomes by training modality and group.

Satisfaction item	PG mean ± SD	SG mean ± SD	MG1 mean ± SD	MG2 mean ± SD	Comparison 1 *p*-value	Comparison 2 *p*-value	Comparison 3 *p*-value
Conventional jaw-model training					PG vs. MG1	PG vs. MG2	MG1 vs. MG2
Clinical case adequacy	4.65 ± 0.49	–	4.24 ± 0.83	4.59 ± 0.51	0.170	0.786	0.274
Model realism	4.53 ± 0.72	–	3.82 ± 0.81	4.18 ± 0.53	**0**.014*	0.092	0.245
Usefulness for manual dexterity development	4.59 ± 0.51	–	4.88 ± 0.33	4.82 ± 0.53	0.150	0.182	0.973
Haptic simulation training					SG vs. MG1	SG vs. MG2	MG1 vs. MG2
Simulation realism	–	4.24 ± 0.44	3.88 ± 0.70	4.41 ± 0.80	0.218	0.218	**0**.029*
Adequacy of simulated clinical cases	–	4.41 ± 0.51	4.65 ± 0.49	4.24 ± 0.66	0.245	0.540	0.099
Graphic size and text adequacy	–	4.41 ± 0.71	4.53 ± 0.51	4.18 ± 0.88	0.786	0.496	0.322
Interface intuitiveness	–	4.35 ± 0.70	4.29 ± 0.77	4.47 ± 0.72	0.892	0.610	0.540

Values are presented as mean ± standard deviation. Between-group comparisons were performed using pairwise Mann–Whitney U tests. The comparison labels are defined separately for each training modality in the shaded rows. “–” indicates that the corresponding group did not complete that training modality. Bold *p*-values indicate statistically significant pairwise comparisons (*p < 0.05). No adjustment for multiple comparisons was applied; statistically significant findings should therefore be interpreted as exploratory.

Following haptic simulation training, mean scores ranged from 3.88 to 4.65. Simulation realism was rated significantly higher in MG2 (4.41 ± 0.80) than in MG1 (3.88 ± 0.70; *p* = 0.029). No statistically significant differences were observed for the remaining satisfaction items (all *p* > 0.05). A non-significant difference was observed for the adequacy of the simulated clinical cases, with higher ratings in MG1 than in MG2 (4.65 ± 0.49 vs. 4.24 ± 0.66; *p* = 0.099).

### 3.5 Motivation

Motivation ratings were high across both training modalities (Table 4). Following conventional jaw-model training, mean scores ranged from 4.59 to 4.82. The highest ratings were observed for motivation to learn, interest in implantology training, and willingness to recommend the session. No statistically significant between-group differences were detected for any motivation item (all *p* > 0.05).

**Table 4:** Motivation outcomes by training modality and group.

Motivation item	PG mean ± SD	SG mean ± SD	MG1 mean ± SD	MG2 mean ± SD	Comparison 1 *p*-value	Comparison 2 *p*-value	Comparison 3 *p*-value
Conventional jaw-model training					PG vs. MG1	PG vs. MG2	MG1 vs. MG2
Helped me better understand theoretical implantology concepts	4.59 ± 0.62	–	4.76 ± 0.44	4.76 ± 0.44	0.518	0.518	1.000
Motivated me to learn more	4.71 ± 0.47	–	4.82 ± 0.39	4.82 ± 0.39	0.563	0.563	1.000
Implantology training is of interest during undergraduate education	4.82 ± 0.39	–	4.82 ± 0.39	4.76 ± 0.44	1.000	0.786	0.786
I would recommend this implantology training session to other students	4.71 ± 0.47	–	4.82 ± 0.53	4.82 ± 0.39	0.433	0.563	0.812
Haptic simulation training					SG vs. MG1	SG vs. MG2	MG1 vs. MG2
Haptic simulation is valuable for dental education	–	4.71 ± 0.47	4.94 ± 0.24	4.82 ± 0.39	0.245	0.563	0.563
Helped me better understand theoretical implantology concepts	–	4.53 ± 0.62	4.65 ± 0.61	4.82 ± 0.39	0.586	0.231	0.540
Motivated me to learn more	–	4.41 ± 0.80	4.53 ± 0.51	4.65 ± 0.61	0.919	0.474	0.474
Future cohorts should have access to haptic simulation technology	–	4.76 ± 0.44	4.88 ± 0.33	4.71 ± 0.47	0.563	0.786	0.394

Values are presented as mean ± standard deviation. Between-group comparisons were performed using pairwise Mann–Whitney U tests. The comparison labels are defined separately for each training modality in the shaded rows. “–” indicates that the corresponding group did not complete that training modality. No adjustment for multiple comparisons was applied.

Following haptic simulation training, mean scores ranged from 4.41 to 4.94. Ratings were particularly high regarding the educational value of haptic simulation and future access to the technology for undergraduate cohorts. No statistically significant between-group differences were observed for any of the evaluated items (all *p* > 0.05).

Within-participant comparisons of the corresponding satisfaction and educational items between training modalities were also performed separately in MG1 and MG2. In MG2, no statistically significant differences were observed between conventional jaw-model and haptic simulation training for any of the seven paired items (all *p* > 0.05). In MG1, perceived clinical case adequacy was rated significantly higher after haptic simulation than after conventional jaw-model training (*p* = 0.035). Motivation to learn showed a numerical difference favoring haptic simulation, although this did not reach statistical significance (*p* = 0.059). No statistically significant differences were observed for the remaining paired items (all *p* > 0.05).

### 3.6 Self-perceived improvement after training

Self-perceived improvement ratings were generally favourable across both training modalities (Table 5). Following conventional jaw-model training, mean scores ranged from 3.65 to 4.76. A statistically significant between-group difference was observed for practical skills, with higher ratings in PG (4.47 ± 0.51) than in MG1 (3.71 ± 0.85; *p* = 0.009). No statistically significant differences were detected for theoretical knowledge, critical thinking, motivation, confidence, or stress management (all *p* > 0.05).

**Table 5:** Self-perceived improvement after practice by training modality and group.

Self-perceived improvement domain	PG mean ± SD	SG mean ± SD	MG1 mean ± SD	MG2 mean ± SD	Comparison 1 *p*-value	Comparison 2 *p*-value	Comparison 3 *p*-value
After conventional jaw-model training					PG vs. MG1	PG vs. MG2	MG1 vs. MG2
Theoretical knowledge	4.18 ± 0.39	–	3.88 ± 0.78	4.18 ± 0.64	0.339	0.919	0.339
Practical skills	4.47 ± 0.51	–	3.71 ± 0.85	4.18 ± 0.81	**0**.009**	0.375	0.106
Critical thinking	4.18 ± 0.64	–	3.76 ± 1.03	4.18 ± 0.81	0.274	0.838	0.231
Motivation	4.76 ± 0.44	–	4.53 ± 0.62	4.47 ± 0.62	0.357	0.218	0.786
Confidence	4.24 ± 0.66	–	3.65 ± 0.93	3.88 ± 0.78	0.073	0.246	0.474
Stress management	4.06 ± 0.75	–	3.88 ± 0.93	4.18 ± 0.81	0.734	0.586	0.375
After haptic simulation training					SG vs. MG1	SG vs. MG2	MG1 vs. MG2
Theoretical knowledge	–	3.76 ± 0.56	3.76 ± 0.44	3.94 ± 0.75	0.838	0.658	0.563
Practical skills	–	3.76 ± 0.75	3.76 ± 0.83	4.00 ± 1.12	0.919	0.306	0.339
Critical thinking	–	4.06 ± 0.56	3.41 ± 0.80	4.00 ± 1.00	**0**.022*	0.838	0.057
Motivation	–	4.65 ± 0.49	4.59 ± 0.51	4.47 ± 0.62	0.786	0.496	0.683
Confidence	–	4.12 ± 0.33	4.00 ± 0.79	3.76 ± 1.09	0.683	0.518	0.658
Stress management	–	4.12 ± 0.70	3.65 ± 0.79	3.82 ± 0.88	0.114	0.433	0.454

Values are presented as mean ± standard deviation. Between-group comparisons were performed using pairwise Mann–Whitney U tests. The comparison labels are defined separately for each training modality in the shaded rows. “–” indicates that the corresponding group did not complete that training modality. Bold *p*-values indicate statistically significant pairwise comparisons (*p < 0.05; **p < 0.01). No adjustment for multiple comparisons was applied; statistically significant findings should therefore be interpreted as exploratory.

Following haptic simulation training, mean scores ranged from 3.41 to 4.65. Critical thinking was rated significantly higher in SG (4.06 ± 0.56) than in MG1 (3.41 ± 0.80; *p* = 0.022). No statistically significant between-group differences were observed for theoretical knowledge, practical skills, motivation, confidence, or stress management (all *p* > 0.05).

Within-participant comparisons of the six corresponding self-perceived improvement domains between conventional jaw-model and haptic simulation training were performed separately in MG1 and MG2 using the Wilcoxon signed-rank test. No statistically significant differences between training modalities were observed for theoretical knowledge, practical skills, critical thinking, motivation, confidence, or stress management in either MG1 or MG2 (all *p* > 0.05).

### 3.7 Harms

No adverse events or harms were reported during either conventional jaw-model training or haptic simulation training.

## 4 Discussion

### 4.1 Accuracy

No statistically significant between-group differences in implant placement accuracy were detected within either training modality. Nevertheless, MG1 showed numerically lower coronal, apical, and angular deviations during conventional jaw-model training than PG and MG2. A similar numerical pattern was observed during haptic simulation training when MG1 was compared with MG2.

These findings suggest that prior exposure to haptic simulation may have influenced subsequent performance; however, the absence of statistically significant differences does not allow a definitive effect of training sequence to be established. The relatively small sample size and limited training exposure may have reduced the ability to detect between-group differences.

In contrast, Zhang et al. (17) reported significantly greater implant placement accuracy in students who received combined virtual and jaw-model training than in those exposed to a single modality, including markedly lower angular deviation in the combined-training group. Differences in training duration, number of repetitions, feedback, and outcome assessment may explain the stronger effects observed in that study.

Previous research suggests that the effectiveness of haptic simulation may depend on repeated practice and structured feedback (2, 3). The present results therefore support the use of haptic simulation as a complementary educational approach rather than as a replacement for conventional jaw-model training.

This interpretation is consistent with recent evidence on students’ acceptance and experiences of haptic simulation. Zadrożny et al. (18) reported favorable student acceptance of immersive virtual reality with haptic simulation, with students supporting its integration alongside conventional training rather than its use as a replacement. Similarly, Baumgartner and Srinivasan (19) found that dental students perceived haptic simulation as a valuable supplementary educational tool while emphasizing the continued importance of conventional phantom-based training. Taken together, these recent findings support a blended educational approach in which haptic simulation complements established preclinical training methods rather than replacing them.

### 4.2 Procedural time

No statistically significant between-group differences were observed in drilling time or total procedure time within either training modality. However, MG1 showed the numerically shortest median drilling time during both conventional jaw-model training and haptic simulation training. During conventional training, median drilling time was 3.10 min in MG1 and 3.47 min in MG2, whereas during haptic simulation it was 0.84 min in MG1 and 1.04 min in MG2.

These numerical differences should be interpreted cautiously because they were small and did not reach statistical significance. They may indicate a possible sequence-related effect, but the present study was not sufficiently powered to establish whether completing haptic simulation first improves procedural efficiency.

Joseph et al. (16) reported significant reductions in drilling and total procedure times after repeated simulator practice. The stronger time-related improvement observed in that study may be explained by repeated exposure to the simulator, whereas the present study evaluated a more limited educational intervention. Further studies with longer training periods and repeated assessments are needed to determine whether haptic simulation produces measurable improvements in procedural efficiency.

### 4.3 Satisfaction

Satisfaction ratings were high for both conventional jaw-model training and haptic simulation training, indicating that both approaches were well accepted by students. This finding is consistent with previous studies reporting positive student perceptions of simulation-based dental education (16, 20, 21).

Differences were observed specifically in perceived realism. PG rated the conventional jaw model as more realistic than MG1, whereas MG2 rated the haptic simulation as more realistic than MG1. Within MG1, the paired analysis also showed higher perceived clinical case adequacy after haptic simulation than after conventional jaw-model training. These findings may reflect differences related to prior exposure and training order; however, because they were limited to individual questionnaire items and no adjustment for multiple comparisons was applied, they should be considered exploratory rather than definitive evidence of a sequence effect. Overall, both conventional jaw-model training and haptic simulation were well accepted as components of undergraduate implantology education.

### 4.4 Motivation

Motivation ratings were consistently high across both training modalities, with no statistically significant between-group differences. Students reported strong interest in implantology training, motivation to continue learning, and support for the use of haptic simulation in future undergraduate cohorts.

These findings are in line with previous studies showing that haptic simulation can enhance engagement and provide a stimulating learning environment, even when objective performance differences are limited (2, 3, 22, 23). The high ratings observed in both modalities also indicate that conventional jaw-model training remains an effective and motivating educational approach.

Because motivation scores were close to the upper limit of the scale, a ceiling effect may have reduced the ability to detect differences between groups. Future studies using more sensitive instruments or qualitative methods may provide a more detailed understanding of how training modality and sequence influence student motivation.

### 4.5 Self-perceived improvement after training

Self-perceived improvement ratings were generally favourable after both conventional jaw-model training and haptic simulation training. Students reported perceived improvement in theoretical knowledge, practical skills, critical thinking, motivation, confidence, and stress management.

After conventional jaw-model training, PG reported higher perceived improvement in practical skills than MG1. After haptic simulation training, SG reported higher perceived improvement in critical thinking than MG1. These findings indicate that self-perceived improvement after training may vary according to both training modality and prior training sequence.

Nevertheless, the significant findings were limited to two isolated questionnaire items and should be interpreted cautiously, particularly because the questionnaires were developed specifically for this exploratory study and had not undergone formal psychometric validation. In addition, no correction for multiple comparisons was applied.

Previous studies have mainly focused on objective technical performance rather than students’ perceptions of improvement after training (16, 24–26). Therefore, the present findings add preliminary information on students’ perceptions of their own learning, but they should not be interpreted as direct evidence of actual competency acquisition. Future research should combine validated student-reported outcome measures with objective performance measures and longer follow-up periods.

### 4.6 Limitations

This study was conducted at a single institution with a relatively small sample size. The small group size limited statistical power to detect potentially meaningful between-group differences; therefore, non-significant findings should not be interpreted as evidence of equivalence between training approaches. In addition, multiple pairwise comparisons were performed without adjustment for multiplicity, increasing the risk of type I error. Accordingly, isolated statistically significant questionnaire findings should be regarded as exploratory and hypothesis-generating. The student-reported questionnaires were developed specifically for this exploratory study and did not undergo formal psychometric validation. Consequently, findings related to satisfaction, motivation, and self-perceived improvement should be regarded as exploratory student perceptions rather than validated measures of these constructs. In particular, self-perceived improvement should not be interpreted as evidence of objectively demonstrated competency acquisition. Future research should combine validated student-reported outcome measures with objective performance measures and longer follow-up periods. Training exposure was limited, and long-term skill retention or transfer to clinical practice was not assessed.

An additional limitation concerns the different methods used to quantify implant-placement accuracy in the two training modalities. Accuracy after conventional jaw-model training was determined by CBCT-based superimposition of the student and reference models, whereas accuracy during haptic simulation was calculated automatically by the simulator. Although both approaches reported analogous coronal, apical, vertical, and angular deviations, differences in measurement procedures, reference definitions, software processing, and measurement error may limit direct quantitative comparability between modalities. Cross-modality comparisons of accuracy should therefore be interpreted cautiously.

## 5 Conclusion

Within the limitations of this randomized pilot study, haptic simulation was a well-accepted complementary tool for undergraduate implantology education. No statistically significant between-group differences were detected in implant placement accuracy or procedural time. However, given the exploratory pilot design and limited sample size, these findings should not be interpreted as evidence of equivalence between training approaches. Simulation-first training showed numerically favorable results for selected accuracy and drilling-time outcomes, although these findings remain exploratory. In addition, multiple pairwise comparisons were performed without adjustment for multiplicity, increasing the risk of type I error. Accordingly, isolated statistically significant questionnaire findings should be considered exploratory and hypothesis-generating.

Satisfaction and motivation were high for both conventional jaw-model and haptic simulation training. Isolated differences in perceived realism and self-perceived improvement after training suggest that training sequence may influence students’ subjective learning experience; however, these exploratory findings should be interpreted cautiously. Larger multicenter studies with repeated training exposure, validated assessment instruments, and participant-level longitudinal analyses are required to determine whether haptic simulation produces sustained improvements in practical skill acquisition and clinical performance.

## Data Availability

The raw data supporting the conclusions of this article will be made available by the authors, without undue reservation.
