# Supplementary material for: Haptic versus conventional training in undergraduate implantology education: a randomized controlled pilot study
Source: Front Oral Health. 2026 Sep 28;7:1938209. doi: 10.3389/froh.2026.1938209 (PMC13654713; doi:10.3389/froh.2026.1938209)
Supplement: Supplementary file 1 [file Table1.docx]

**Supplementary Material**

*Questionnaires used to evaluate satisfaction, motivation, and self-assessed competencies*

This supplementary file provides the questionnaire items used in the study. Items were answered using Likert-type scales as indicated in each table.

# Supplementary Table S1. Satisfaction questionnaire

| **Item** | **Question** | **Training modality** | **Response scale** |
| --- | --- | --- | --- |
| 1 | The clinical case presented was appropriate and consistent with the theoretical content of Implantology. | Conventional jaw-model training | 1 = strongly disagree; 5 = strongly agree |
| 2 | The surgical resin model used for implant placement seemed realistic. | Conventional jaw-model training | 1 = strongly disagree; 5 = strongly agree |
| 3 | Laboratory sessions are useful for developing manual dexterity. | Conventional jaw-model training | 1 = strongly disagree; 5 = strongly agree |
| 4 | The simulation appeared realistic in terms of case design and instrument handling. | Haptic simulation practice | 1 = strongly disagree; 5 = strongly agree |
| 5 | The clinical case presented was appropriate and aligned with the theoretical content. | Haptic simulation practice | 1 = strongly disagree; 5 = strongly agree |
| 6 | The simulator interface was intuitive and ergonomically suitable. | Haptic simulation practice | 1 = strongly disagree; 5 = strongly agree |
| 7 | The graphic size and text readability of the material were adequate. | Haptic simulation practice | 1 = strongly disagree; 5 = strongly agree |

# Supplementary Table S2. Motivation questionnaire

| **Item** | **Question** | **Training modality** | **Response scale** |
| --- | --- | --- | --- |
| 1 | This practical session helped me better understand theoretical concepts of Implantology, such as the drilling sequence. | Conventional jaw-model training | 1 = strongly disagree; 5 = strongly agree |
| 2 | Performing a clinical implantology case in the laboratory motivated me to learn more. | Conventional jaw-model training | 1 = strongly disagree; 5 = strongly agree |
| 3 | Implantology laboratory practice is of interest during undergraduate training. | Conventional jaw-model training | 1 = strongly disagree; 5 = strongly agree |
| 4 | I would recommend the implantology laboratory practice to other students. | Conventional jaw-model training | 1 = strongly disagree; 5 = strongly agree |
| 5 | Haptic simulation practice is valuable for dental education. | Haptic simulation practice | 1 = strongly disagree; 5 = strongly agree |
| 6 | This practice helped me understand theoretical implantology concepts, such as the drilling sequence. | Haptic simulation practice | 1 = strongly disagree; 5 = strongly agree |
| 7 | Performing an implantology case using a haptic simulator motivated me to learn more. | Haptic simulation practice | 1 = strongly disagree; 5 = strongly agree |
| 8 | Future cohorts should have access to haptic simulation technology during their training. | Haptic simulation practice | 1 = strongly disagree; 5 = strongly agree |

# Supplementary Table S3. Self-perceived improvement after training

**Question:** After completing this training, to what extent do you think you have improved in the following areas?

| **Item** | **Competency** | **Context** | **Response scale** |
| --- | --- | --- | --- |
| 1 | Theoretical knowledge | Self-perceived improvement after training | 1 = not at all; 5 = significantly |
| 2 | Practical skills | Self-perceived improvement after training | 1 = not at all; 5 = significantly |
| 3 | Critical thinking | Self-perceived improvement after training | 1 = not at all; 5 = significantly |
| 4 | Motivation | Self-perceived improvement after training | 1 = not at all; 5 = significantly |
| 5 | Confidence | Self-perceived improvement after training | 1 = not at all; 5 = significantly |
| 6 | Stress management | Self-perceived improvement after training | 1 = not at all; 5 = significantly |

**Note.** The satisfaction and motivation questionnaires used a five-point agreement scale. The self-assessment questionnaire used a five-point perceived improvement scale.
